# Application of the Principles of Green Chemistry for the Development of a New and Sensitive Method for Analysis of Ertapenem Sodium by Capillary Electrophoresis

**DOI:** 10.1155/2019/1456313

**Published:** 2019-01-02

**Authors:** Tahisa Marcela Pedroso, Ann Van Schepdael, Hérida Regina Nunes Salgado

**Affiliations:** ^1^UNESP-Univ Estadual Paulista, Faculdade de Ciências Farmacêuticas, Araraquara, São Paulo, Brazil; ^2^KU Leuven-University of Leuven, Department of Pharmaceutical and Pharmacological Sciences, Pharmaceutical Analysis, Leuven, Belgium

## Abstract

An innovative method is validated for the analysis of ertapenem sodium by capillary electrophoresis using potassium phosphate buffer 10 mM pH 7 and 15 kV voltage, in the concentration range of 70 to 120 *μ*g mL^−1^. Ertapenem had a migration time of 3.15 minutes and the linearity curve was y = 2281.7 x - 24495 with a R^2^ = 0.9994. Thus, we propose a routine analysis method that meets the principles of green analytical chemistry for the routine analysis of ertapenem sodium by capillary electrophoresis.

## 1. Introduction

Capillary electrophoresis is a versatile separation technique, which can be used for a wide range of substances. The technique consists in the migration of electrically charged species, present in an electrolytic solution inside a capillary, to which an electric field is applied, generating a current in its interior. The technique of capillary electrophoresis has been used for the separation of drugs.

In February of 2017, in Geneva, the World Health Organization (WHO) published its first ever list of antibiotic-resistant “priority pathogens,” a catalogue of 12 families of bacteria that pose the greatest threat to human health. Antibiotic resistance has been increasing and treatment options have been rapidly lost. The list highlights the threat of Gram-negative bacteria that are resistant to multiple antibiotics. Ertapenem sodium (ERTM) is a *β*-lactam antimicrobial from the carbapenem class. This class of drugs has activity against Gram-positive, Gram-negative, aerobic, and anaerobic bacteria.

**Figure 1:**
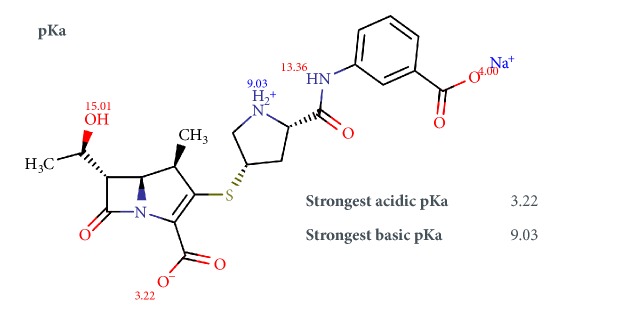
Chemical structure of ertapenem sodium with pKa calculation by* Chemicalize*. *∗*Source: https://chemicalize.com/#/calculation.

ERTM is a polar and ionizable compound (Figure 1) that is distinguished from the other carbapenems by its anionic side chain composed of a benzoate group. The substituted benzoic acid target is crucial to maintain its antibacterial spectrum; moreover, it increases the molecular weight and lipophilicity. The carboxylic acid unit, which is ionized at physiological pH, results in a net negative charge. As a result, ERTM is highly bound to plasma proteins, allowing the convenience of being administered only once daily [1]. Furthermore, it is more stable to renal dehydropeptidase, not requiring the addition of any enzyme inhibitor as with other drugs of this group [2].

Ionizable species represent the majority of the compounds analyzed in the pharmaceutical industry. ERTM is a molecule that presents acidic, basic, and amphoteric pKas. The pKa values were calculated using the online platform* Chemicalize* that yielded the strongest acidic pKa at 3.22 and the strongest basic pKa at 9.03.

Capillary electrophoresis (CE) is an important technique for analysing many pharmaceutical and biopharmaceutical substances. The CE technique has been widely used for the analysis of small molecule drugs, excipients, and counter ions in pharmaceuticals, for determination of impurities and for the analysis of proteins, glycoproteins, complex carbohydrates, liposaccharides, DNA therapeutics, and virus particles. CE is one of the most powerful techniques applicable as a method of choice for the characterization and quality control of biomolecules in the biopharmaceutical industry. With such a strongly growing industry, there is an inevitable demand for advanced analytical techniques, which could be applied as sensitive and reliable tools in development and quality control of these products to ensure their safety and efficiency [3–6].

Currently, there is a growing demand for faster, more economical and environmentally friendly analytical methods. Among the analytical separation techniques, CE is considered a “green” alternative due to its low vapour pressure, low sample volume, and reduced analysis time, which consequently allows the reduction of solvent use and reduction of generated waste. It thus contributes substantially to the efficient use of electric energy and further enables the development of methods without the use of toxic solvents, making it safe for analysts. For these properties, it has been presented as an ecofriendly technique [7, 8].

The capillary electrophoresis technique has been suggested for routine analysis in the frame of the quality control of drugs in their pharmaceutical formulation [9–12]. CE has also been presented as a green alternative for food analysis [8]. With this, laboratories are beginning to consider CE as a standard routine procedure for the separation of samples [13].

Green chemistry is a current topic that has been much neglected in different areas by the academic community and is globally encouraged by researchers and companies with environmental awareness. Analytical methods which prioritize environmental sustainability have been presented in the literature as ecofriendly method; ecological method; green analytical method; environmentally friendly method ([7, 14–23]; Tótoli et al., 2014). Effective and reliable analytical methods, which can quantify the antimicrobial content, are essential for evaluating drug quality. Thus, this work presents a capillary electrophoresis method for routine evaluation of ertapenem sodium lyophilized powder for injection.

## 2. Experimental

### 2.1. Apparatus

The method was carried out on a P/ACE™ MDQ (Beckman Coulter™) capillary electrophoresis system with UV detector and a fused silica capillary with internal diameter of 75 *μ*m, outer diameter 375 *µ*m, effective length of 30 cm, and total length 40 cm. The used electrolyte was 10 mM sodium phosphate buffer at pH 7. An analytical balance model SECURA2250-1S (Sartorius™, Goettingen-Germany) was used. The chemicals used were ertapenem sodium 98.8% (ID number 1407011333e) and ertapenem sodium lyophilized powder for injection (lot EB004C1) both kindly donated by Merck Sharp & Dohme™. Capillary rinsing was performed with NaOH solution at the concentrations of 1 M and 0.1 M and 0.1 M HCl as well as purified water obtained through Milli-Q™ Plus equipment (Millipore™ USA). The reagents used for the degradation were 0.01 M hydrochloric acid (Qhemis™), 0.01 M sodium hydroxide (Cinetica™), and 0.03% m/m hydrogen peroxide (Vetec™). All solutions were filtered through a nylon membrane with 0.45 *μ*m pore size and 47 mm diameter (Millipore™) and were degassed in an ultrasonic bath, model 2510E-MT (Branson™, Danbury-CT USA).

### 2.2. Methodology

The capillary electrophoresis method was performed using 10 mM sodium phosphate buffer at pH 7 as electrolyte; prior to each analysis the capillary was washed with this electrolyte for 2 min. Analyses were performed using 15 kV voltage, electric current 48 *µ*A, and an injection time of 5 seconds (Pressure 0.5 psi). The cartridge temperature was 25°C and the detector wavelength was set at 214 nm. The diluent solution, the electrolyte, the solutions used to promote degradation, and the adjuvants sodium hydroxide and sodium bicarbonate were evaluated as blank solution, without any trace of ERTM, to evaluate possible interfering peaks during the analysis. The method was validated in accordance with the guidelines [24, 25]. The evaluated parameters were linearity, limit of quantitation, limit of detection, selectivity, precision (repeatability and intermediate precision), accuracy, and robustness.

In order to evaluate the robustness of the method, a factorial matrix of Plackett Burman was used. In this mathematical model it is possible to evaluate small alterations to parameters simultaneously. This factorial matrix has been successfully applied to the evaluation of robustness in many analytical techniques ([26–32]; Pedroso, Salgado, 2014)

### 2.3. Solutions

An ERTM Reference Chemistry Standard (RCS) stock solution was prepared by transferring 10 mg of ERTM RCS to a 10 mL volumetric flask, which was filled with ultrapure water to obtain a concentration of 1000 *μ*g mL^−1^. Aliquots from this stock solution were transferred to 10 mL volumetric flasks, the volumes of which were completed with water, to obtain working solutions of 70, 80, 90, 100, 110 and 120 *µ*g mL^−1^. Five vials of ERTM lyophilized powder for injection were weighed, and the average weight was calculated. The contents of these vials were mixed. The stock solution from ERTM lyophilized powder was prepared in the same way as ERTM RCS stock solutions described above.

### 2.4. Electrolyte Preparation

For the preparation of the 10 mM potassium phosphate buffer solution pH 7, 136 mg of dibasic potassium phosphate and 40 mg of monobasic potassium phosphate were dissolved in 100 mL purified water. When necessary, the pH was adjusted to 7 using 6 M phosphoric acid or 10 M potassium hydroxide as recommended by the Brazilian Pharmacopeia [33].

## 3. Results and Discussion

Preliminary tests were performed to evaluate the parameters that, together, could provide a reliable method. The definition of capillary length is important, since the migration time is influenced by the effective length (the length of the injection point to the detection point), but also by the total capillary length and the separation voltage. It was decided to work initially with a capillary of 40 cm total length and 30 cm effective length. If necessary, this length could be adjusted, however it appeared to be adequate.

Different buffer solutions at different pHs were tested as electrolyte. Generally, the buffering systems are effective in a pH range corresponding to their pKa, plus or minus one pH unit. With this, some options of buffer solutions were tested as electrolyte.

In fused silica capillaries, the working pH may range from 2 to 11; however, one should also consider the molecule's stability in that pH range and its own pKa to then choose the appropriate electrolyte. That is why, when separation involves molecules with an acid-base character, the molecule's electrophoretic mobility depends on the electrolyte pH. In this case, the effective mobility term, which incorporates the product of the electrophoretic mobility of species in equilibrium and the distribution of the relative concentrations of each species at that pH, must be considered.

**Figure 2:**
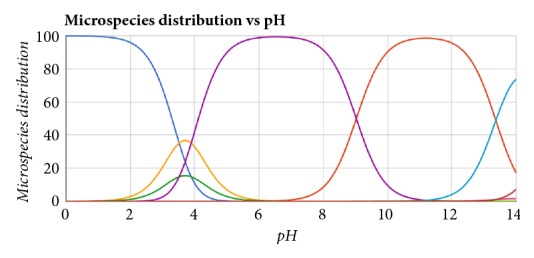
Distribution of ERTM microspecies as visualized with* Chemicalize*. The curves of the microspecies are assigned according to the following colour codes: dark blue: ERTM^+^; yellow: ERTM neutral, green: ERTM neutral; purple: ERTM^−^; orange: ERTM^2-^; light blue: ERTM^3-^; red: ERTM^4-^. *∗*Each color at microspecies distribution diagram represents the protonation states that can be checked on the* online platform Chemicalize *https://chemicalize.com/#/calculation.

Therefore, pH control is advisable, and the choice of a suitable buffer solution has direct implications for the optimization of the separation. In this way,* Chemicalize* online software was used to evaluate the distribution of microspecies versus pH and, by doing so, defining the electrolyte that is in the best pH range to be used.  Figure 2 shows this microspecies distribution for ERTM. Each color in the microspecies distribution diagram represents the different protonation states calculated for the molecule and allows us to view the major protonation form at a determined pH.

In the analysis of the distribution of microspecies for ertapenem sodium at each pH, the possibility of working at a pH around 7 or 11 was verified. Therefore, phosphate and borate buffers were chosen for the initial tests.

Borate buffer is one of the most used buffers in capillary electrophoresis; it is preferred because it has large ions with low mobility and can be used in high concentrations without the disadvantage of generating excessive heat. However, it has the disadvantage of absorbing more in the UV region compared to the phosphate buffer. In addition, it is not advisable to use an electrolyte with a pH close to the working pH limit, in order to preserve the capillary and to guarantee the results' repeatability, since highly alkaline pH promotes the dissolution of the silica present in the capillary. Thus, borate buffer pH 10 and phosphate buffer pH 7 were chosen for the analysis of ertapenem sodium. As expected, the ERTM peak using borate buffer pH 10 was distorted, with a front tail probably because the anion molecule mobility is different from the electrolyte anion mobility. In contrast, the phosphate buffer showed a symmetrical peak and was therefore chosen for further method development.

A high electrolyte concentration and applied voltage can compromise the separation due to the excess heat caused by the Joule effect. Joule heating results in the formation of a temperature gradient and generates a current inside the capillary, causing the mixing of the already separated bands and resulting in the dispersion of the peak. This effect can be minimized by the application of suitable voltages and the use of lower concentration buffers coupled with good temperature control. However, buffer solutions with low concentrations may increase the adsorption tendency of the molecules to the capillary wall and peak tailing can be observed in the electropherogram. Moreover, at low concentrations, the electroosmotic flow can become erratic, which hinders the repeatability of migration times and consequently impairs the identification and quantification of the substance under analysis. The high electrical resistance of the capillary allows the application of high electric fields, as it generates a minimum heating; in addition, the capillary shape provides efficient dissipation of the heat generated. The advantage of using high voltages is a gain in resolution and efficiency, as well as a decrease in analysis time [34].

The electrolyte concentration and equipment voltage were adjusted in order to obtain a current not greater than 50 *μ*A, a range in which the equipment was previously validated for use, although, theoretically speaking, it has the capacity to work up to 300 *μ*A. Thus, the concentration of the phosphate buffer was set at 10 mM with a voltage of 15 kV. The temperature in the cartridge containing the capillary was controlled at 25°C.

The “dead” migration time was verified by using the blank solution that was the electrolyte itself. Sodium hydroxide and sodium bicarbonate adjuvants, as well as the solutions used to promote drug degradation without any trace of ERTM, were used in order to evaluate any other possible peaks during the analysis. The degrading solutions present a small baseline oscillation at 2 min migration time. At this migration time, the small peak in red present in the electropherogram of Figure A1 (supplementary material) corresponds to the 0.03% m/m hydrogen peroxide solution used to promote forced drug degradation.

**Figure 3:**
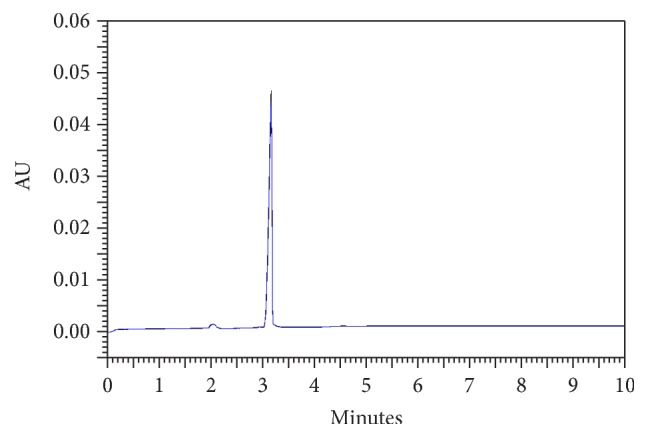
Comparison of ERTM electropherograms RCS (blue) versus ERTM lyophilized powder for injection (black) by the capillary electrophoresis method.

Thus, it has been found that there is no interference of the degrading solutions and/or the adjuvants contained in the pharmaceutical formulation for the quantification of ERTM by the proposed method, since the migration time of ERTM is 3.2 min. The qualitative analysis was performed by comparing the electropherograms of ERTM RCS versus ERTM lyophilized powder for injection that showed the same migration time (Figure 3).

### 3.1. System Suitability Test (SST)

The system suitability test was conducted to evaluate the system resolution and repeatability to ensure that the complete testing system was suitable for the intended application. In order to obtain the required data, ten solutions of ERTM reference standard at a concentration of 100 *μ*g mL^−1^ were prepared and analysed by CE. The parameters such as corrected peak area, migration time, plate number (N), and relative standard deviation (%RSD) were calculated and their acceptance limits were analysed according to Bose, 2014, in the same way as chromatography [35] (Table 1).

### 3.2. Calculation of ERTM Average Content in Lyophilized Powder for Injection

The average content of ERTM lyophilized powder for injection is calculated by the dosage of the chemical versus the reference sample, in triplicate, at concentrations of 100 *μ*g mL^−1^. The sample solution readings were evaluated at the wavelength of 214 nm. The concentration of ertapenem sodium in the sample is calculated by (1) and its percentage content by (2). The average content found was 99.94% with an RSD of 0.85%.

(1) CS=ASCRSARS

(2) CS%=CSCT×100

where *C*_*s*_ is the sample concentration (*μ*g mL^−1^), *C*_*s*_% is the percentage content, *C*_*Rs*_ is the concentration of chemical reference standard (*μ*g mL^−1^), *A*_*s*_ is the sample corrected peak area, *A*_*Rs*_ is the reference standard corrected peak area, and *C*_*t*_ is the theoretical concentration of ERTM in the sample (*μ*g mL^−1^).

### 3.3. Method Validation

This method was validated according to the International Council on Harmonization guidelines ICH, 2005 and Harmonized Guidelines for Single Laboratory Validation of Methods of Analysis from IUPAC [25, 36] for linearity, selectivity, accuracy, precision, robustness, detection limit, and quantification limit.

#### 3.3.1. Linearity

The linearity was evaluated by regression analysis of ERTM. The analytical curve was constructed by plotting the concentration versus the average of the corrected peak area values of each ERTM RCS concentration. The assay was performed in triplicate on three different days. The regression lines were calculated by the least-squares method (Figure A2 in the supplementary material). Statistical evaluation was made by ANOVA (Table 2). The homoscedasticity of the data was investigated by plotting the residuals (Figure A3 in the supplementary material), as recommended by RDC #166, which provides for the validation of analytical methods and other measures [24]. The standard residues are less than 2% and therefore the model is considered suitable for use. The values were reported as the average %RSD of the calibration curves.

#### 3.3.2. Detection Limit (LOD) and Quantification Limit (LOQ)

The LOD and LOQ were determined using the calibration curve made in triplicate and calculated as 3 *σ*/S and 10 *σ*/S, respectively, where “S” is the slope of the calibration curve and “*σ*” is the standard deviation of the response.

#### 3.3.3. Precision

Repeatability* (intraday precision) *and intermediate precision* (interday precision): *the repeatability was studied by performing seven determinations of the sample at the median concentration of the calibration curve. The solutions were prepared and analyzed the same day under the same experimental conditions. The intermediate precision was evaluated by the average percentage RSD obtained for the triplicate analysis on different days.

#### 3.3.4. Accuracy

Accuracy was obtained via a recovery assay, in which known quantities of ERTM reference standard were added to a known quantity of the sample. The recovery was investigated at 3 different concentrations, R1, R2, and R3, equivalent to 80, 100, and 120% of the average concentration (Table 3). Each simulated sample (R1, R2, and R3) was assayed in an independent trial. The analysis was performed in triplicate and the percentage recovery (R%) was calculated according to Equation (3) of the* Association of Official Analytical Chemists*.

(3) $$\begin{array}{c} R\%=\frac{Cf-Cu}{Ca}\times 100 \end{array}$$

*where*

*Cf* is the total drug concentration measured after addition of the standard;

*Cu* is the total drug concentration in the formulation;

*Ca* is the standard concentration added to the formulation.

Table 4 summarizes the results of the method validation and percentage content determination.

#### 3.3.5. Selectivity by Study of Forced Degradation

The stress study was determined by subjecting an ERTM lyophilized powder for injection (100 *µ*g mL^−1^) to accelerated degradation by alkaline, acid, neutral, oxidative, thermal, and photolytic stress, in order to evaluate the effect of degradation products on the quantitation of ERTM. Acid hydrolysis was performed in 0.01 M HCl, base hydrolysis in 0.01 M NaOH, and for the oxidative solution stress study, a sample solution of ERTM was prepared using 0.03% m/m hydrogen peroxide in water as diluent. The solutions were evaluated for 5 days. Concurrently, also a control sample (ERTM dissolved in water only) was injected for comparison. The photolytic stress of ERTM was achieved by exposing a sample of ERTM lyophilized powder for injection to UV light of 254 nm. A sample of ERTM lyophilized powder for injection, which was wrapped in aluminum foil, was used as the dark control so that there were no interferences. The authentic sample and the dark control were placed in separate glass Petri dishes and spread across the dish to give a thickness of no more than 3 mm, in accordance with ICH guidelines. Both samples were exposed to the UV light for 5 days. For the solid-state thermal stress, an aliquot of ERTM lyophilized powder for injection was stressed by storage at 50°C and analyzed hourly. The results are shown in Table 5.

#### 3.3.6. Robustness

Robustness is evaluated by making small changes to the parameters to demonstrate that the validity of the method is maintained. Plackett-Burman factorial design was chosen to evaluate these parameters simultaneously, whereby 15 experiments are held with 7 parameters ranging in the upper and lower levels.

The Tables 6, 7, and 8 show the factorial combination used in the Plackett-Burman test, letters A to G represent the selected parameters. The numbers 1 to 15 account for the th number of experiments (2n + 1). Whereby n is the number of parameters, (0) corresponds to the normal pre-set parameters in the process and the numbers (1) and (-1) are the upper and lower levels of these parameters.

The robustness was determined from injections of standard versus sample solutions containing 100 *μ*g mL^−1^ ERTM under the same experimental conditions. The influence of each parameter was determined by comparing the average of the dosage performed in triplicate assays corresponding to normal ranges to the average of the dosage corresponding to the modified levels. The average effect of each variable is the average difference between the observations made at the modified levels and those made at the optimum level. The deviation of each of those parameters was calculated by using the methodology of Youden and Steiner [37, 38]. Equation (4) gives an illustration on how this methodology evaluates the effect of changing parameter A: Buffer Concentration. The other parameters were evaluated similarly.

(4) $$\begin{array}{c} \sqrt{2S}>\left|\;DA\;\right| \end{array}$$

*where*

(5) S=27DA2+DB2+DC2+DD2+DE2+DF2+DG2

The deviation of each changed parameter (DA, DB, DC, etc.) ought to be less than the value resulting from √2S to infer that the effects obtained with the variations of the parameters are not significant. The method is robust for all of the selected parameters (Table 9).

## 4. Conclusion

There are many applications of the capillary electrophoresis technique. Some studies involve the monitoring of environmental pollutants [39]. It has also been used for metal determination [40], as well as for food analysis [41, 42] and drug analysis [43–47]. In this study, we used ERTM for the development of a protocol for validation of the capillary electrophoresis method based on the principles of green chemistry, as an option for routine drug analysis.

The system suitability test was performed prior to validation to ensure that the selected parameters were adequate. The proposed capillary electrophoresis method for the routine quantification of ERTM was validated for the parameters selectivity, linearity, precision, accuracy, limit of quantification, and limit of detection, as recommended in the international guidelines [25].

The ERTM migration time was 3.2 min, thereby providing rapid drug determination. The selectivity was determined by subjecting sodium ertapenem samples to stress conditions by forced degradation in alkaline, acidic, neutral, oxidative, and photolytic media. No products were seen that could interfere with drug quantification.

The linearity was evaluated by construction of a calibration curve in triplicate, which presented the equation y = 2281.7 x – 24495, R^2^ 0.9994. Statistical analysis of variance (ANOVA) was performed and the results showed that there are no significant deviations of linearity and, therefore, the method is linear in the range of 70-120 *µ*g mL^−1^.

The average content obtained at three different concentration levels within the linear range should be evaluated in triplicate and present an RSD <2%. The content of ertapenem sodium in the analyzed samples was 99.94%, RSD 0.85%.

The method was evaluated according to the repeatability and the intermediate precision. The levels obtained for the triplicate assays of ERTM RCS* versus* ERTM in lyophilized powder for injection presented an RSD < 2%.

The robustness was evaluated by the Plackett-Burman factorial model, in which small changes of the analytical parameters occur simultaneously. No altered effect presented a significant result when compared to the reference value, thus demonstrating that the method is robust.

The accuracy was evaluated by the recovery test of a known amount of analyte added to the sample, as determined by the Association of Official Analytical Chemists [24, 48]. The method accuracy was proven by the recovery test, since the average percentage recovered was 100.59%, RSD 1.09%.

Thus, an alternative method to chromatographic methods was developed, with the advantages of using reduced sample amounts, having low analysis time and not using any kind of organic or toxic solvent, and being safe for the analyst and not generating toxic waste to be treated. Capillary electrophoresis is considered a green analytical technique because it does not cause damage to the environment. The proposed electrophoretic method presented linearity, precision, accuracy, and robustness according to the current guidelines; therefore, it can be used for the quantitative analysis of ertapenem sodium in the pharmaceutical industry.

Just like chromatography, capillary electrophoresis is a separation technique which can be used for a wide range of substances. Although not used as much as chromatographic techniques, capillary electrophoresis stands out due to the high separation power with small amounts of sample and reagents, low residue generation, low toxicity, low cost per analysis, and reduced analysis time when compared to HPLC. Capillary electrophoresis is a complementary separation technique for routine analysis and has gained interest due to its important green character.

## Figures and Tables

**Table 1:** Parameters evaluated in the system compliance analysis, for determination of ERTM by capillary electrophoresis.

	**Corrected peak area**	**Migration time (min)**	**Plate number**	**Asymmetry**
1	200747	3.17	11120	0.85
2	206731	3.20	10947	0.83
3	206201	3.19	11045	0.84
4	205646	3.19	10895	0.85
5	203986	3.19	10824	0.84
6	205936	3.21	10624	0.84
7	207729	3.23	10537	0.85
8	200253	3.23	11046	0.84
9	205686	3.23	10782	0.84
10	204768	3.20	10868	0.84

SD	2617.67	0.02	197.46	0.01
RSD (%)	1.28	0.70	1.82	0.69

RSD = relative standard deviation.

**Table 2:** Analysis of variance of calibration curve of ertapenem sodium RS by capillary electrophoresis.

**Source of variation **	**Degree of freedom**	**Sum of squares**	**Variability**	**F calculated**	**F critical**
**Between concentration**	5	27348000160.07	5469600032.01	1993.04*∗*	3.11
**Linear regression**	1	27315622241.54	27315622241.54	9953.42*∗*	4.75
**Deviation of linearity**	4	32377918.53	8094479.63	2.95	3.26
**Residue**	12	32932159.12	2744346.59	........	.......
**Total **	17	27380932319.19	........	........	.......

*∗* Significant at p <0.05.

**Table 3:** Accuracy of the capillary electrophoresis method, as obtained via a recovery assay.

	**Sample** ^**a**^ µ**g mL**^**-1**^	**Standard** ^**a**^ µ**g mL**^**-1**^	**Concentration **µ**g mL**^**-1**^	%
**Sample**	70	-----	70	-----
**R1**	70	10	80	80%
**R2**	70	30	100	100%
**R3**	70	50	120	120%
**Standard**	-----	70	70	-----

^a^The analysis was performed in triplicate.

**Table 4:** Results of capillary electrophoresis method validation and percentage content determination.

**PARAMETERS**	**RESULTS**
Content of ERTM	99.94%
Linearity	y = 2281.7x – 24495 R^2^ = 0.9994 (70 to 120 *µ*g mL^−1^)
Repeatability^a^	RSD = 1.62%
Intermediate precision^b^	1st day 102.84%; 2nd day 99.83% and 3rd day 99.15% - RSD = 0.85%
Accuracy^b^	100.59%, RSD = 1.09%
LOD	0.77 *µ*g mL^−1^
LOQ	2.32 *µ*g mL^−1^
Recovery	100.59%

^a^Seven determinations; ^b^Average of three determinations.

**Table 5:** Study of forced degradation.

	**Time**	**Degradation (**%**)**
Neutral	2 days	24.39%
0.01M NaOH	3 hours	20.85%
0.01M HCl	15 min	23.43%
0.03% H_2_O_2_	45 min	23.24%
Thermal 50°C	3 hours	21.12%
UVC_254_ light	5 days	22.47%

**Table 6:** Factors and Levels of variability using the experimental model of Plackett-Burman.

**Parameter**	**Unit**	**Limit**	**(-1)**	**(0)**	**(** **1)**
(A) Buffer Concentration	m*M*	1	9	10	11
(B) Voltage	kV	1	14	15	16
(C) Wavelength	nm	1	213	214	215
(D) Injection Time	s	1	4	5	6
(E) Rinsing of capillary	min	1	1	2	3
(F) Temperature of cartridge	°C	1	24	25	26
(G) Temperature of sample storage	°C	1	24	25	26

**Table 7:** Robustness test using the experimental model of Plackett-Burman.

**Analytical Parameter**	**Factorial Combination**														
	**1**	**2**	**3**	**4**	**5**	**6**	**7**	**8**	**9**	**10**	**11**	**12**	**13**	**14**	**15**
A	1	1	1	0	1	0	0	0	-1	-1	-1	0	-1	0	0
B	0	1	1	1	0	1	0	0	0	-1	-1	-1	0	-1	0
C	0	0	1	1	1	0	1	0	0	0	-1	-1	-1	0	-1
D	1	0	0	1	1	1	0	0	-1	0	0	-1	-1	-1	0
E	0	1	0	0	1	1	1	0	0	-1	0	0	-1	-1	-1
F	1	0	1	0	0	1	1	0	-1	0	-1	0	0	-1	-1
G	1	1	0	1	0	0	1	0	-1	-1	0	-1	0	0	-1

A–G: selected factors; 1–15: N (number of experiments) = 2n + 1, where n = number of factors; −1, 0, +1: levels for the factors.

**Table 8:** Factors evaluated in the experimental model of Plackett-Burman.

**Analytical Parameter**	**Factorial Combination**														
	**1**	**2**	**3**	**4**	**5**	**6**	**7**	**8**	**9**	**10**	**11**	**12**	**13**	**14**	**15**
(A) Buffer Concentration	11 m*M*	11 m*M*	11 m*M*	10 m*M*	11 m*M*	10 m*M*	10 m*M*	10 m*M*	9 m*M*	9 m*M*	9 m*M*	10 m*M*	9 m*M*	10 m*M*	10 m*M*
(B) Voltage	15 kV	16 kV	16 kV	16 kV	15 kV	16 kV	15 kV	15 kV	15 kV	14 kV	14 kV	14 kV	15 kV	14 kV	15 kV
(C) Wavelength	214 nm	214 nm	215 nm	215 nm	215 nm	214 nm	215 nm	214 nm	214 nm	214 nm	213 nm	213 nm	213 nm	214 nm	213 nm
(D) Injection Time	6 s	5 s	5 s	6 s	6 s	6 s	5 s	5 s	4 s	5 s	5 s	4 s	4 s	4 s	5 s
(E) Rinsing of capillary	2 min	3 min	2 min	2 min	3 min	3 min	3 min	2 min	2 min	1 min	2 min	2 min	1 min	1 min	1 min
(F) Temperature of cartridge	26°C	25°C	26°C	25°C	25°C	26°C	26°C	25°C	24°C	25°C	24°C	25°C	25°C	24°C	24°C
(G) Temperature of sample storage	26°C	26°C	25°C	26°C	25°C	25°C	26°C	25°C	24°C	24°C	25°C	24°C	25°C	25°C	24°C

**Table 9:** Results of robustness for ERTM analysis by CE.

**Analytical Parameter **	**(-1)**	**Content of test (-1) (**%**)**^**a**,**b**^	**(** **1)**	**Content of test (** **1) (**%**)**^**a**,**b**^
(A) Buffer Concentration	9 m*M*	100.49 − 100.20 = |0.29|	11 m*M*	100.84 − 100.22 = |0.62|
(B) Voltage	14 kV	100.66 − 100.03 = |0.63|	16 kV	100.02 − 101.04 = |1.03|
(C) Wavelength	213 nm	100.35 − 100.34 = |0.01|	215 nm	100.62 − 100.44 = |0.17|
(D) Injection Time	4 s	100.31 − 100.39 = |0.08|	6 s	101.03 − 100.03 = |1.01|
(E) Rinsing of capillary	1 min	100.68 − 100.02 = |0.66|	3 min	100.49 − 100.57 = |0.08|
(F) Temperature of cartridge	24°C	101.23 − 100.46 = |0.23|	26°C	100.73 − 100.33 = |0.40|
(G) Temperature of sample storage	24°C	100.15 − 100.54 = |0.40|	26°C	100.23 − 100.82 = |0.59|

^a^Subtraction of average contents in normal conditions and average contents in the altered conditions ^b^Reference criteria calculated |1.31| for test (1) and |0.80| for test (-1).

## Data Availability

The data used to support the findings of this study are included within the article.

## Supplementary Materials

Supplementary Materials
** Figure A1:** Blank solution: electrolyte, adjuvants, and degrading solutions without any traces of ERTM, analysed with the capillary electrophoresis method.** Figure A2:** Linearity curve of ERTM in CE.** Figure A3:** Homoscedasticity of the CE method.** Figure A4:** Neutral degradation (room temperature at 25°C), after 120 hours.** Figure A5:** Photolytic stress (UVC_254_ light).** Figure A6:** Acid degradation (0.01M HCl), after 120 hours.** Figure A7:** Alkaline degradation (0.01M NaOH), after 36 hours.** Figure A8:** Oxidative degradation (0.3% H_2_O_2_), after 24 hours.** Figure A9:** Thermal stress (oven at 50°C), after 1 hour.
