# Supplementary material for: Application of the Principles of Green Chemistry for the Development of a New and Sensitive Method for Analysis of Ertapenem Sodium by Capillary Electrophoresis
Source: Int J Anal Chem. 2019 Jan 2;2019:1456313. doi: 10.1155/2019/1456313 (PMC6334326; doi:10.1155/2019/1456313)

**Supplementary material - Appendice A**

**Fig. A1-** Blank solution: electrolyte, adjuvants and degrading solutions without any traces of ERTM, analysed with the capillary electrophoresis method


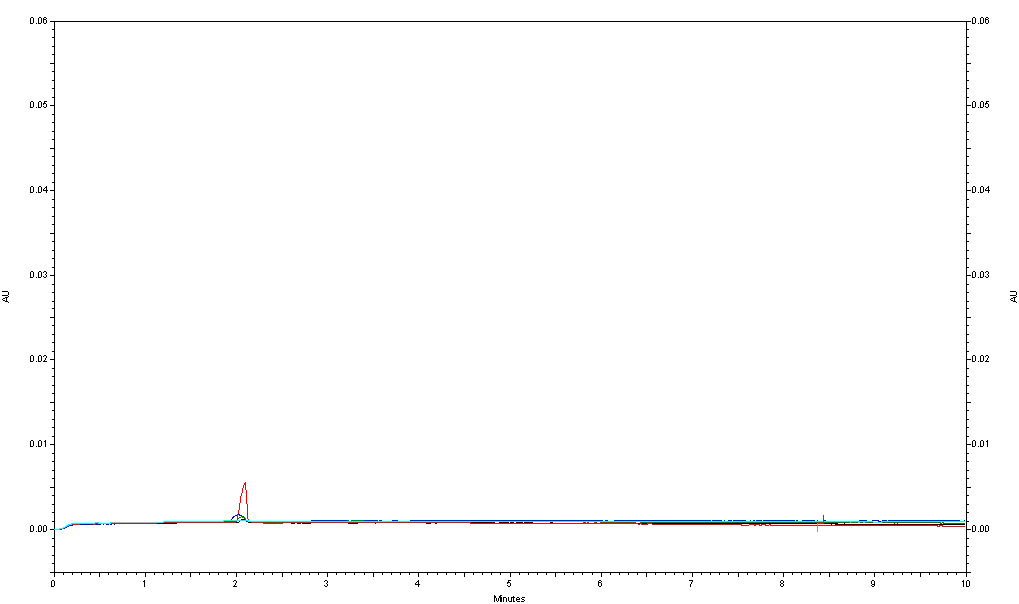


**Fig. A2-** Linearity curve of ERTM in CE


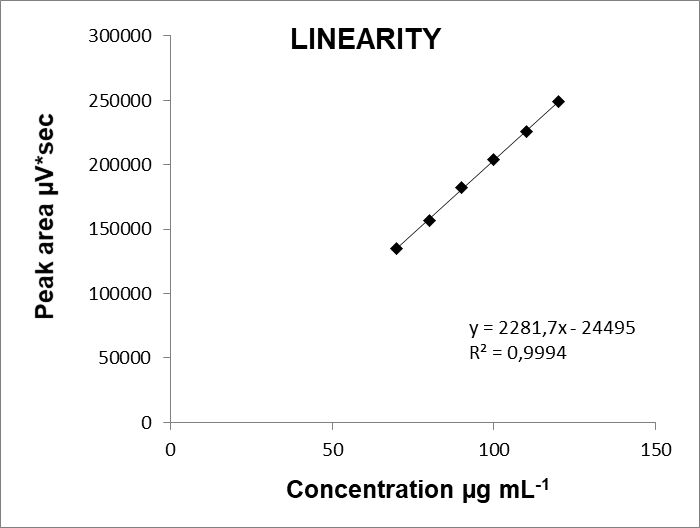


**Fig. A3-** Homoscedasticity of the CE method


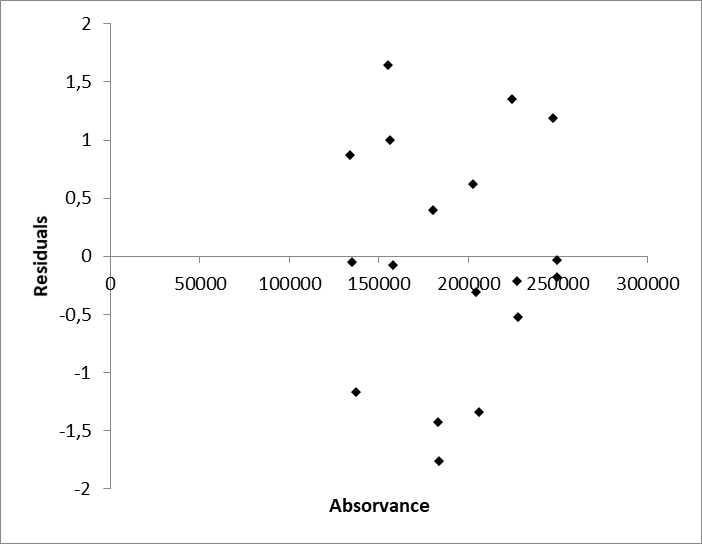


**Fig. A4-** Neutral degradation (room temperature at 25 ºC), after 120 hours


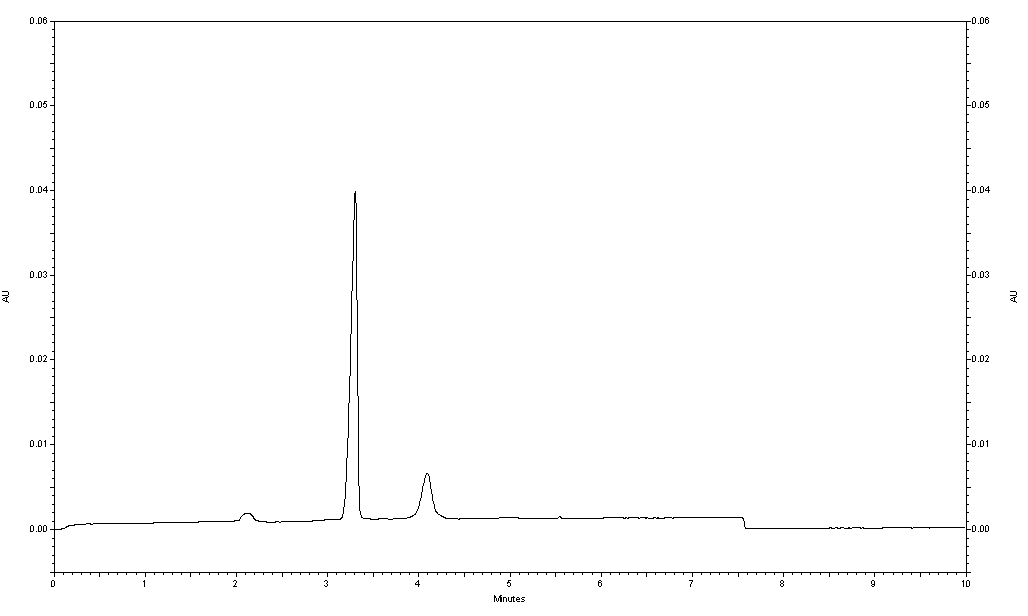


**Fig. A5-** Photolytic stress (UVC_254_ light)


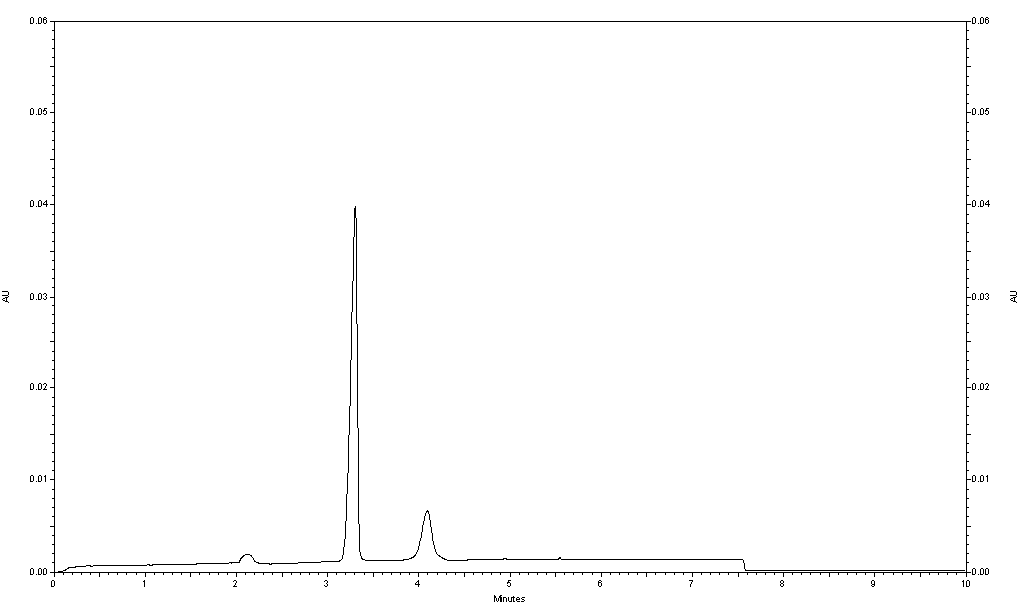


**Fig. A6-** Acid degradation (0.01M HCl), after 120 hours


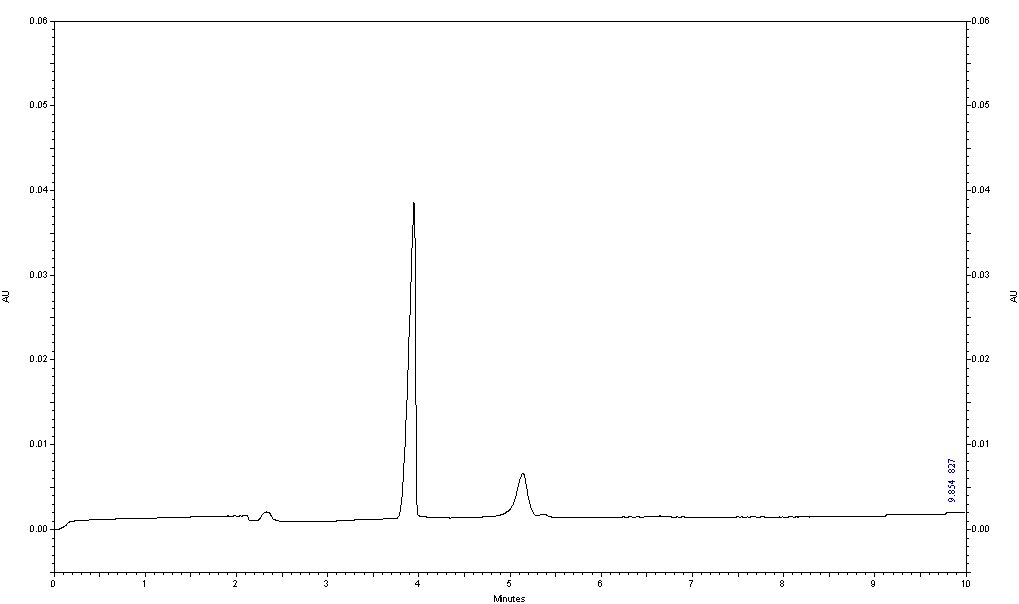


**Fig. A7-** Alkaline degradation (0.01M NaOH), after 36 hours


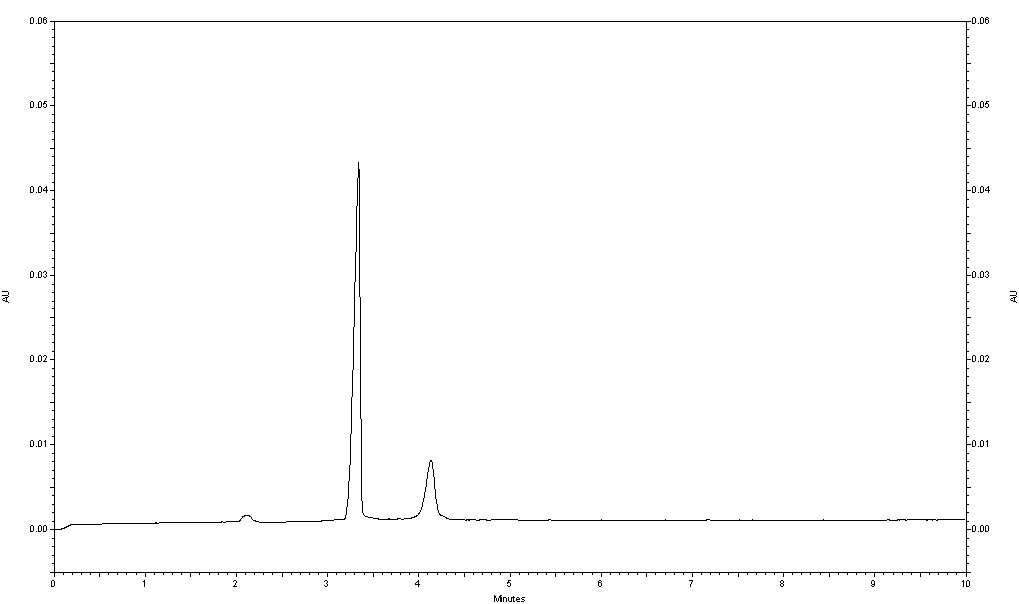


**Fig. A8-** Oxidative degradation (0.3%H_2_O_2_), after 24 hours


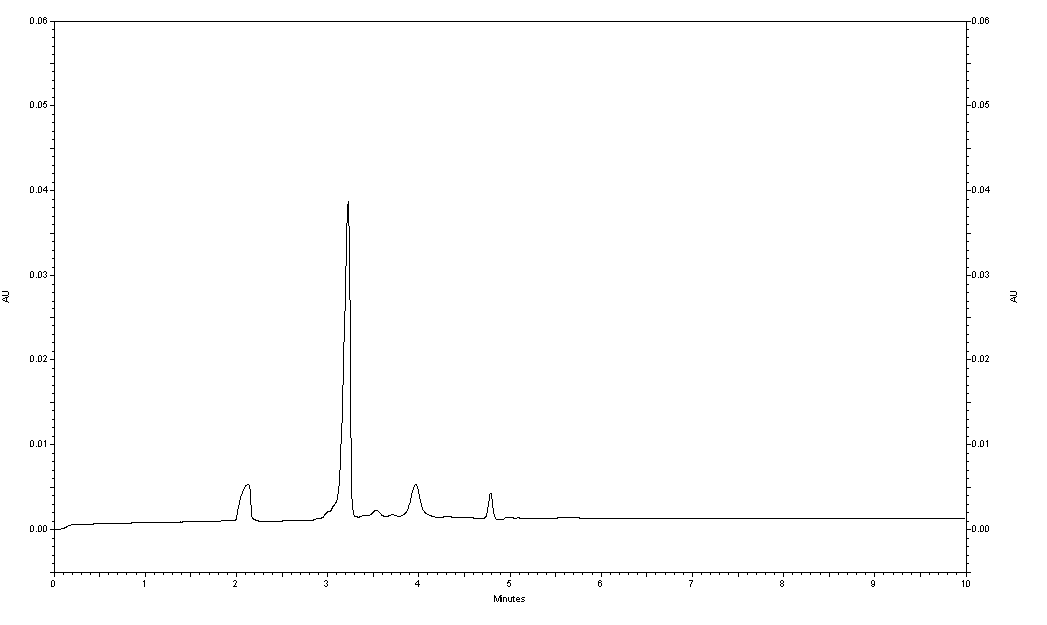


**Fig. A9-** Thermal stress (oven at 50 ºC), after 1 hour


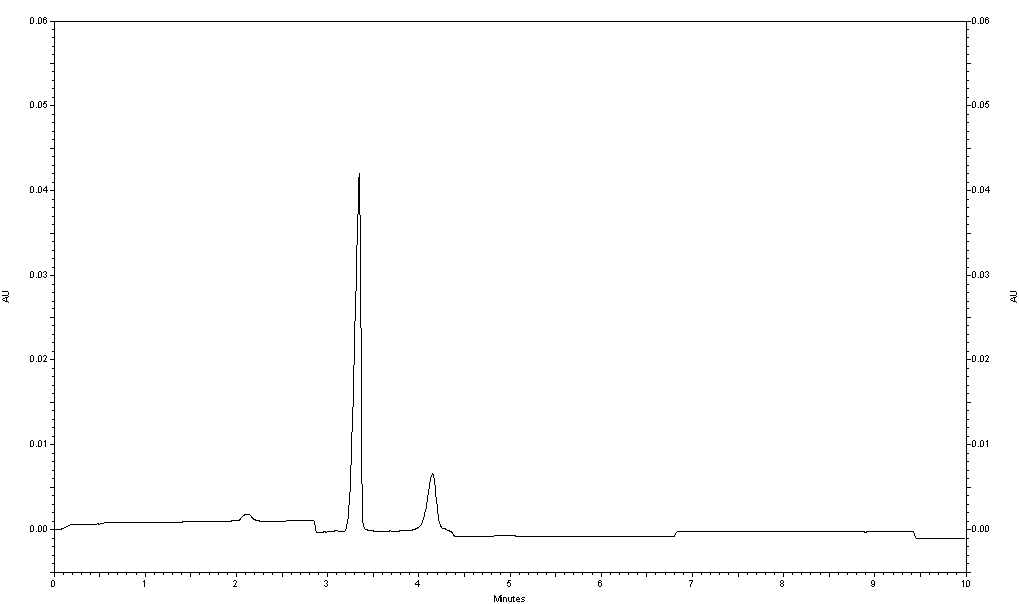

Supplement: Supplementary Materials — Figure A1: Blank solution: electrolyte, adjuvants, and degrading solutions without any traces of ERTM, analysed with the capillary electrophoresis method. Figure A2: Linearity curve of ERTM in CE. Figure A3: Homoscedasticity of the CE method. Figure A4: Neutral degradation (room temperature at 25°C), after 120 hours. Figure A5: Photolytic stress (UVC254 light). Figure A6: Acid degradation (0.01M HCl), after 120 hours. Figure A7: Alkaline degradation (0.01M NaOH), after 36 hours. Figure A8: Oxidative degradation (0.3% H2O2), after 24 hours. Figure A9: Thermal stress (oven at 50°C), after 1 hour. [file 1456313.f1.docx]
